# Molecular Modeling of ALK L1198F and/or G1202R Mutations to Determine Differential Crizotinib Sensitivity

**DOI:** 10.1038/s41598-019-46825-1

**Published:** 2019-08-06

**Authors:** Yu-Chung Chuang, Bo-Yen Huang, Hsin-Wen Chang, Chia-Ning Yang

**Affiliations:** 1 Department of Life Sciences, National University of Kaohsiung, Kaohsiung, Taiwan; 2 Scientific Multi-Simulation Center, National University of Kaohsiung, Kaohsiung, Taiwan

**Keywords:** Computational biology and bioinformatics, Molecular modelling

## Abstract

Anaplastic lymphoma kinase (ALK) is a receptor tyrosine kinase that has been recognized as a therapeutic target for EML4-ALK fusion-positive nonsmall cell lung cancer (NSCLC) treatment using type I kinase inhibitors such as crizotinib to take over the ATP binding site. According to Shaw’s measurements, ALK carrying G1202R mutation shows reduced response to crizotinib (IC_50_ = 382 nM vs. IC_50_ = 20 nM for wild-type), whereas L1198F mutant is more responsive (IC_50_ = 0.4 nM). Interestingly, the double mutant L1198F/G1202R maintains a similar response (IC_50_ = 31 nM) to the wild-type. Herein we conducted molecular modeling simulations to elucidate the varied crizotinib sensitivities in three mutants carrying L1198F and/or G1202R. Both L1198 and G1202 are near the ATP pocket. Mutation G1202R causes steric hindrance that blocks crizotinib accessibility, which greatly reduces efficacy, whereas mutation L1198F enlarges the binding pocket entrance and hydrophobically interacts with crizotinib to enhance sensitivity. With respect to the double mutant L1198F/G1202R, F1198 indirectly pulls R1202 away from the binding entrance and consequently alleviates the steric obstacle introduced by R1202. These results demonstrated how the mutated residues tune the crizotinib response and may assist kinase inhibitor development especially for ALK G1202R, analogous to the ROS1 G2302R and MET G1163R mutations that are also resistant to crizotinib treatment in NSCLC.

## Introduction

Anaplastic lymphoma kinase (ALK) is a member of the insulin receptor protein–tyrosine kinase (RTK) superfamily, and has been recognized as an efficacious therapeutic target for the treatment of non-small cell lung cancer (NSCLC) because of its effect on the EML4-ALK fusion protein that occurs in patients with NSCLC^1,2^. The entire ALK protein structure includes an extracellular portion for ligand binding, a transmembrane segment, and an intracellular portion comprised of a juxtamembrane (JM) segment, a protein kinase domain, and a carboxyl terminal tail. In the basal condition, the ALK kinase domain remains in a uniquely intermediate state between its active and inactive forms. Specifically, the DFG motif adopts a DFG-in orientation, which is a common feature in active kinases^3^. F1271 points inward, which makes the ATP site available, and meanwhile, the aligned C1182, I1171, F1271, and H1247 residues form the R-spine that is usually assembled in the active state of kinase^4,5^. Yet, despite these two active state features, the A-loop adopts an autoinhibitory pose so that the entrance of the peptide substrate binding is blocked, resulting in the inactive state of ALK. To activate the ALK kinase function, the extracellular domain binds with a ligand such as midkine^6^ or pleiotrophin^7^, and dimerization of ALK is induced, which is followed by the phosphorylation of Y1278 on the A-loop by a partner ALK protein kinase domain. This generates a conformation change of the A-loop, and exposes the peptide substrate binding site, which turns on the phosphorylation function^8–11^.

Because ALK kinase dysregulation is involved in numerous diseases, including anaplastic large cell lymphomas, lung cancer, and neuroblastomas, many efforts have been made to develop ALK kinase inhibitors^12–18^; crizotinib^19^ and alectinib^20^ are among these. Crizotinib was approved by the FDA in 2011 for the purpose of treating ALK-positive NSCLC. Despite diseases being highly responsive to crizotinib treatment, roughly one in four patients tend to develop drug resistance within 1 to 2 years. According to the measurement by Friboulet *et al*., several drug-resistant mutations, such as G1202R (IC_50_ = 221 nM), S1206Y (IC_50_ = 124 nM), and G1269A (IC_50_ = 130 nM), are adjacent to the ATP/inhibitor-binding site in the kinase domain, cause steric hindrance to the inhibitors, but enable resumption of ALK activity (wild-type’s IC_50_ = 16 nM)^21^. By contrast, mutation L1198F, located near the ATP/inhibitor-binding site, enhances the sensitivity to crizotinib, achieving an IC_50_ = 0.4 nM compared with the WT’s IC_50_ = 20 nM as measured by Shaw *et al*.^22^. Moreover, Shaw *et al*. also determined that the L1198F/G1202R double mutant restores the sensitivity to crizotinib, achieving IC_50_ = 31 nM, which outperforms the IC_50_ = 382 nM measured for G1202R mutant. It is notable that mutation site G1202R is located in the solvent front of ALK kinase and confers remarkable resistance to numerous ALK inhibitors^23^. Moreover, G1202R in ALK is analogue to ROS1 G2302R and MET G1163R mutations and all these three exhibit drug resistance in NSCLC treatment. Therefore, understanding the drug resistance mechanism caused by G1202R in ALK certainly benefits drug developed for NSCLC.

**Figure 1:**
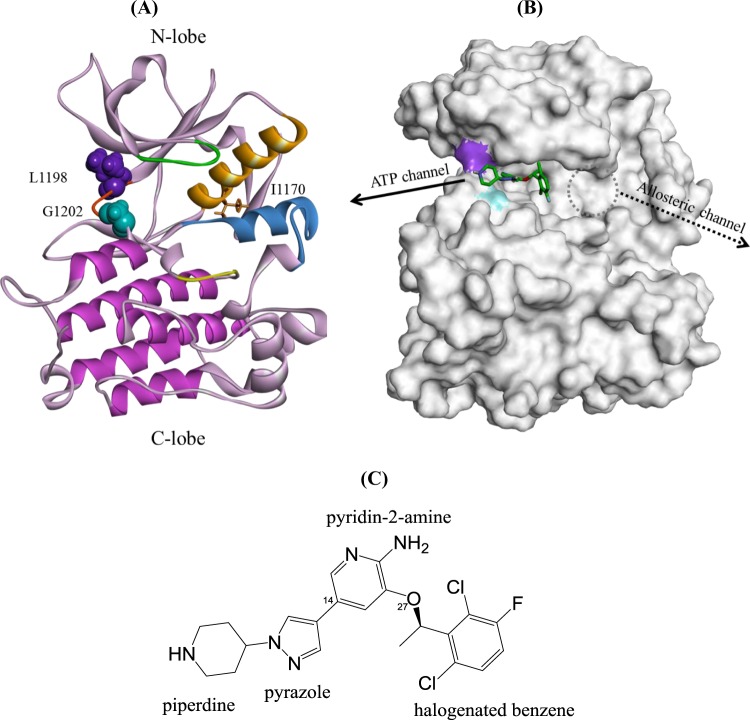
Structure of ALK kinase domain. (**A**) Two mutation sites in this study specified in the ALK. I1170 is also specified for it is used as a reference point in the umbrella sampling in a latter section. (**B**) The crizotinib-bound ALK structure generated in this work to indicate that the binding site is in the cleft between the N- and C-lobes. (**C**) Crizotinib structure.

Figure 1(A) illustrates a structure of ALK kinase domain with the L1198 and G1202 residues specified. The red segment between E1197 and G1202 is the hinge that plays an essential role in maintaining the relative movement between the N-lobe and C-lobe. Figure 1(B) depicts the surface presentation of a crizotinib-bound ALK complex structure where the purple and blue spots are L1198 and G1202, respectively. Figure 1(C) identifies the crizotinib structure with four ring moieties, namely piperidine, pyrazole, pyridine-2-amine, and halogenated benzene. Kinase inhibitors are divided into two types. Type I kinase inhibitors target kinases in the active state. They occupy the ATP pocket where the conserved DFG motif adopts a DFG-in orientation with phenylalanine pointing inward to spare the ATP binding site and also to participate in the R-spine assembly, which keeps the kinase domain in the proper movement to serve the enzyme function. A type II kinase inhibitor interacts with kinases in the inactive state in which DFG phenylalanine points outward and shrinks the ATP pocket, which generates an allosteric pocket in the back. The two types of kinase inhibitors have two types of binding channels^24^. Type I takes the ATP channel, whereas type II takes the allosteric channel. Crizotinib binds to the ATP pocket and is categorized as type I. ALK kinase is in the inactive state in its basal state, but its ATP pocket is available for the type I kinase inhibitors crizotinib and ceritinib^17^. In Fig. 1(B), the ATP channel is indicated by a solid-line arrow. Although the allosteric pocket is blocked by DFG phenylalanine and is not available in ALK, we circled the possible position and pinpointed the corresponding access channel with a dashed-line arrow for the purpose of comparison.

In this study we aimed to answer three questions: (i) how the mutation G1202R causes drug resistance to crizotinib, (ii) how mutation L1198F is more crizotinib-sensitive and superior to the WT, and (iii) how the double mutant L1198F/G1202R returns crizotinib sensitivity to a level similar to the WT’s. Recently, the role played by the binding affinity in drug development has often been criticized for it alone may not always serve as a sole descriptor to indicate drug efficacy^25^. On the other hand, there has been growing evidence to show that binding kinetics correlates better than binding affinity to drug efficacy^26^. Prior to our molecular modeling on the ALK systems, we have postulated that the replaced R1202 in G1202R mutant shall contribute stabilization toward the bound crizotinib, because as indicated in Fig. 1(A) G1202 is in the solvent front and the longer side chain of R1202 certainly provides more contact than G1202 does. Accordingly, the binding affinity of crizotinib in G1202R mutant should be better than that in WT and this introduces a conflict in the observed drug resistance by G1202R replacement. To resolve this conflict, we performed molecular dynamics (MD) simulations on the apo WT, G1202R mutant, L1198F mutant, and L1198F/G1202R mutant to investigate the minute conformation variance near the crizotinib binding pocket entrance so that we could correlate the observed drug accessibility to the varied IC_50_ values. Crizotinib-bound state of WT and the three mutants were also studied to evaluate their binding free energies. Moreover, umbrella sampling^27^ using the equilibrated structures extracted from the MD simulation trajectories for ALK-crizotinib complexes as initial structures was conducted to characterize the dissociation processes of crizotinib from the WT and the three mutants. On the basis of the obtained kinetic and thermodynamic observations, we provided valuable information applicable to drug development of ALK inhibitors.

## Material and Methods

### System setup

AMBER 12.0 software package^28^, assisted by ff99SB force field^29^, was utilized for this MD study. Ligand parameters were from the general AMBER force field (GAFF)^30^ and the Antechamber program. Moreover, the ligand atoms’ partial charges were obtained with electrostatic potential calculations at the HF/6-31G* level^31^ and assigned using AM-BCC protocol. The initial structure of the apo WT was retrieved from the X-ray crystallography solved structure in PDB code 3L9P^32^. With respect to the apo L1198F, G1202R, and L1198F/G1202R mutants, their initial structures were constructed through manual replacement of the mutated amino acid residues on the last conformation of the WT after the 50-ns production process. For the initial structures of the crizotinib-bound complexes, we used the crystal structure solved for the ALK-crizotinib complex in PDB code 5AAA^22^ as a template to superimpose over the last conformations of the apo WT, L1198F, and L1198F/G1202R trajectories after the 50-ns production procedure. Sodium ions were added to neutralize the studied systems: five ions for the apo WT, five ions for the apo L1198F mutant, four ions for the apo G1269R mutant, four ions for the apo L1198F/G1202R mutant, five ions for the WT–crizotinib complex, five ions for the L1198F–crizotinib complex, four ions for the G1202R-crizotinib complex, and four ions for the L1198F/G1202R–crizotinib complex. LEaP module was used to assign hydrogen atoms and protonation states of ionizable amino acid residue at neutral pH state. Each studied system was solvated in a cubic TIP3P water box with a 10 Å minimum solute-to-wall distance^33^ and the added water molecules underwent an energy minimization process comprising of the steepest descent algorithm (7500 steps) and the conjugate gradient algorithm (7500 steps) with a nonbonded cutoff of 8.0 Å. Furthermore, the same energy minimization process was carried out for another two times to relax the amino acid side chains (by restraining protein backbone atoms) and to relax the whole solvated system. For each studied system, we took the structure of the very end of the aforementioned three-staged energy minimization procedure as the initial structure for consecutive MD simulations. A standard protocol covering gradual heating (from 0 to 300 K in 60 ps and at 300 K for 40 ps), density equilibrium (at 300 for 100 ps), and equilibration (at 300 K for 800 ps) in isothermal isobaric ensemble setup (NTP with pressure of 1 atm and temperature of 300 K) was adopted prior to the production MD. As to the three ALK–crizotinib complex systems, each underwent a 50-ns simulation for conformation collection taken every 100 ps and for the subsequent binding free energy calculation (where only the last 10 ns trajectory was considered) and structural analysis. As to the four apo systems, we carried out two independent 50-ns production procedures and collected snapshots at a 100 ps interval for structural evaluation.

All the nonbonding interactions, such as short-range electrostatics and van der Waals interactions, within a cutoff distance 8 Å were considered. As to the long-range electrostatic interactions, the particle-mesh-Ewald method was applied^34^. All bonds involved in hydrogen atom to their equilibrium lengths were constrained by SHAKE algorithm^35^.

### Binding free energy calculations

One hundred snapshots were extracted from the last 10 ns of the WT–crizotinib, L1178F–crizotinib, G1202R–crizotinib, and L1198F/G1202R–crizotinib trajectories to determine their ALK–crizotinib Δ*G*_bind_ calculations. The MM/GBSA method has been taken as an accepted method to calculate the binding free energy between the receptor and its ligand^36,37^. The equation below is adopted:

$${\rm{\Delta }}{G}_{bind}={G}_{ALK-crizotinib}-[{G}_{ALK}+{G}_{crizotinib}]$$

That is, the free energy difference between the complex and the sum of ALK and crizotinib was considered as the binding free energy. Moreover, each *G*_molecule_ term on the right-hand side of the equation is decomposed as follows:

$${G}_{molecule}=\langle {E}_{{\rm{MM}}}\rangle +\langle {G}_{{\rm{solvation}}}^{{\rm{polar}}}\rangle +\langle {G}_{{\rm{solvation}}}^{{\rm{nonpolar}}}\rangle -TS,$$

Herein, the symbol $\langle \ldots \rangle$ denotes the average for structures collected from an MD trajectory. The free energy *G*_molecule_ term is composed of four parts: the gas-phase free energy *E*_MM_, the polar solvation free energy ${G}_{{\rm{solvation}}}^{{\rm{polar}}}$, the nonpolar solvation free energy ${G}_{{\rm{solvation}}}^{{\rm{nonpolar}}}$, and the entropy term −*TS*. Furthermore, the gas-phase free energy is to be calculated by the internal energy *E*_internal_, the electrostatic energy *E*_electrostatic_, and van der Waals energy *E*_vdW_.

$$\langle {E}_{{\rm{MM}}}\rangle =\langle {E}_{{\rm{internal}}}\rangle +\langle {E}_{{\rm{electrostatic}}}\rangle +\langle {E}_{{\rm{vdW}}}\rangle ,$$

The polar solvation term is calculated using the Generalized Born model whereas the nonpolar solvation term is calculated with the surface-tension proportionality constant γ = 0.00542 kcal mol^−1^ Å^−2^ and the nonpolar free energy for a point solute β = 0.92 kcal mol^−1^ using the equation

$${G}_{{\rm{solvation}}}^{{\rm{nonpolar}}}=\gamma A+\beta ,$$

where the solvent-accessible surface area, *A*, is varied by the molecule and calculated by the program. Moreover, the entropy term *TS* attributed by degree of freedom changes, including translational, rotational, and vibrational terms of the solute molecules, is estimated by normal mode analysis (NMA)^38^ using AMBER14’s nmode module. To save computational cost, 30 snapshots evenly extracted from the 40–50 ns production MD trajectories were used for the entropy calculations.

### PMF calculation

The PMF calculation was achieved with umbrella sampling method^27,39^ by collecting multiple overlapping biasing potentials along the ATP-pocket dissociation pathway as the reaction coordinate^40–43^. WHAM^44^ was used to build the free energy profile along the reaction coordinate. Our reaction coordinate was set as the separation distance between the crizotinib C14 atom (pinpointed in Fig. 1(C)) and the ALK I1170 Cα atom (indicated in Fig. 1(A)). A separation distance ranging from 0 to 20 Å was used for the dissociation path, and the reaction coordinate was divided into 50 continuous windows. Each window considered a harmonic biased potential ${u}_{i}=\frac{1}{2}{k}_{i}{(r-{r}_{i})}^{2}$ with the force constant *k*_*i*_ of 10 kcal/mol·Å^2^. The term *u*_*i*_ is the biased potential in window *i*, *r* is the current position of reaction coordinate, and *r*_*i*_ is the reference position in window *i*. Each simulation set used a 0.4-Å window length and for each window, we performed a 10-ns MD run to collect conformations with the temperature set at 300 K.

## Results and Discussion

### Binding energy analysis

**Figure 2:**
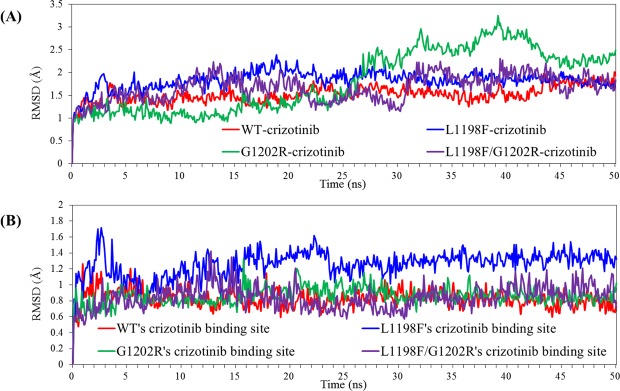
Time evolution of RMSD values with respect to coordinates of heavy atoms of ALK and the crizotinib molecule in the form of ALK-crizotinib complexes. (**A**) RMSD generated by considering all the heavy atoms of the entire ALK-crizotinib complex. (**B**) RMSD generated by considering only the heavy atoms within the 7 Å boundary of the bound crizotinib.

To investigate the energetic contribution influenced by the minute conformational variance caused by mutated amino acid residues, we estimated the binding free energies using MM/GBSA for crizotinib-bound complexes. Figure 2(A) presents the root-mean-square deviation (RMSD) values of the heavy atoms of ALK and crizotinib along the 50-ns production duration for the four ALK-crizotinib complexes. The red curve depicting WT–crizotinib complex reached a stable state after 5 ns, fluctuating within 1.2 and 1.8 Å; the blue curve for L1198F–crizotinib complex became stable after 15 ns, fluctuating within 1.5 and 2.0 Å; the green curve for G1202R-crizotinib complex showed great magnitude indicating some fragments in great flexibility; as for the L1198F/G1202R–crizotinib complex, a jump was evident around 32 ns, and after the jump the purple curve became stable. To investigate the structural mobility of G1202R-crizotinib complex, we calculated different sets of RMSD values by excluding different segments of ALK and concluded that the N-terminal N1093-I1116 segment and the C-terminal D1389-E1400 segment contributed the flexibility. As shown in Fig. S1(A) in Supporting Information, two black parts in ALK structure indicate the locations of these two loose-end segments which are not in close contact with the core ALK structure. Figure S1(B) includes two RMSD curves for the whole G1202R-crizotinib complex (green curve) and the complex with deletion of the two aforementioned segments (green dashed curve) which is obviously stable within the entire production duration. In Fig. S1(C), we plotted this green dashed curve with the other three ALK-crizotinib complexes’ RMSD curves, and the fluctuating magnitudes are very alike. In Fig. 2(B) we plotted the RMSD values by considering only the heavy atoms within the 7 Å boundary of the bound crizotinib. The red, green, and purple curves for WT-, G1202R-, and L1198F/G1202R-crizotinib, respectively, reached a stable state less than 15 ns, whereas the blue curve for L1198F-crizotinib reached a stable state less than 25 ns. All these four curves suggested the structural stability around the binding pocket. In the following, we consider the last 10-ns trajectory for each complex by collecting 100 snapshots at a 100-ps interval to calculate ΔG and analyze structures.

**Figure 3:**
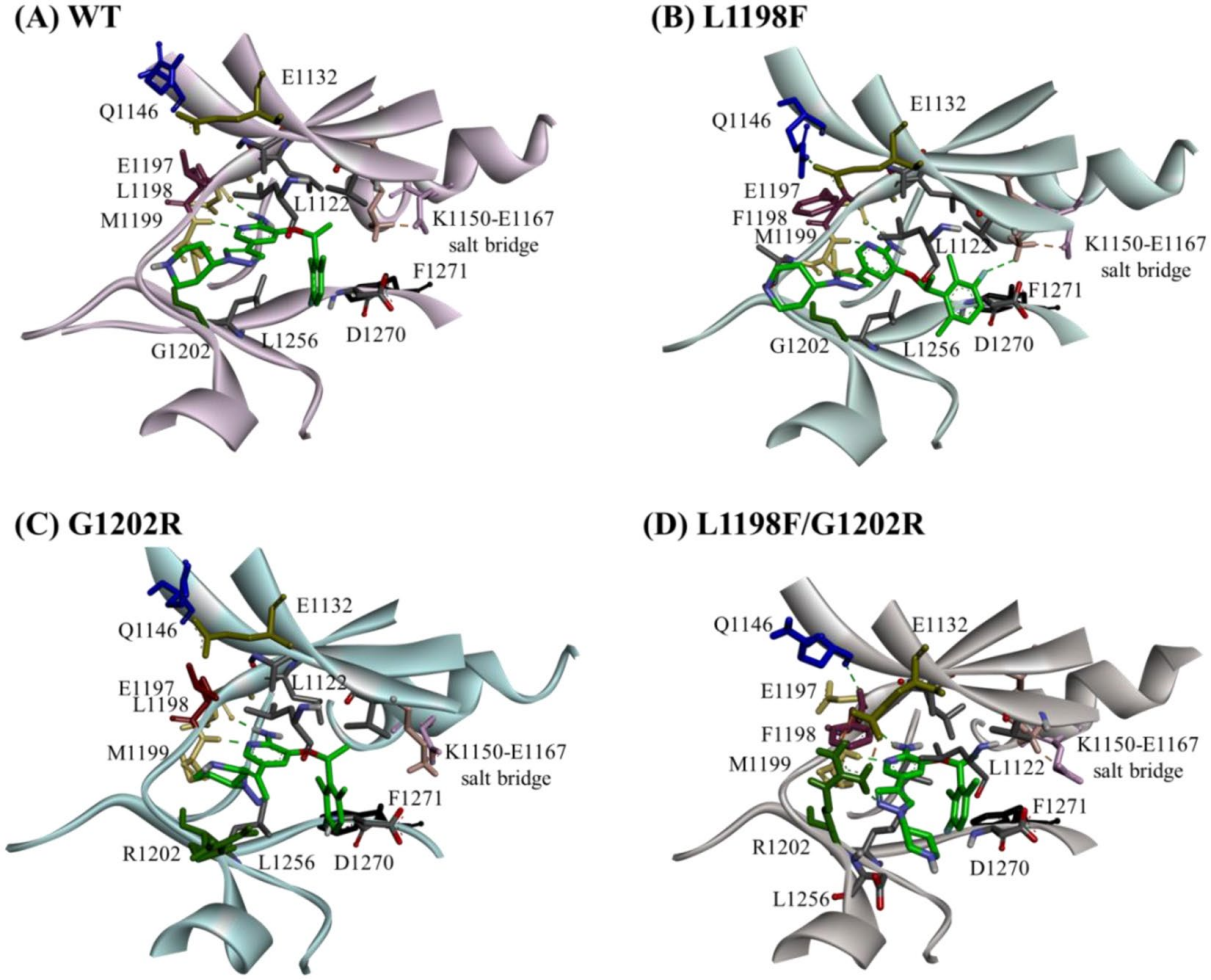
Crizotinib binding modes in ALKs: (**A**) WT, (**B**) L1198F mutant, (**C**) G1202R mutant and (**D**) L1198F/G1202R mutant. Crizotinib (green) and several important residues are shown in stick. L1198 and F1198 are colored in purple; G1202 and R1202 are colored in dark green; L1122 is colored in cyan; E1132 is colored in olive; Q1146 is colored in blue; L1150 is colored in light salmon; E1167 is colored in light pink. Hydrogen bonds and salt bridges are shown in green dash lines and pink dish lines, respectively.

As indicated in Fig. 3(A) for WT–crizotinib, the inhibitor is accommodated in a cleft between the N- and C-lobes, and is in a bent shape with the O27 atom (see Fig. 1(C)) as a pivotal point. The pyridine-2-amine ring moiety occupies the adenine pocket, approaches the ALK hinge, and forms two hydrogen bonds with E1197 and M1199 backbones. That is, crizotinib uses its pyridine nitrogen atom to link to M1199’s backbone amino hydrogen atom, and uses amine hydrogen to link to E1197’s backbone carbonyl oxygen atom. The piperidine ring protrudes toward the solvent front. Moreover, L1256 from the C-lobe and L1122, V1130, A1148, K1150, L1196, and G1202 from the N-lobe engage in hydrophobic interactions to clamp the inhibitor. For L1198F–crizotinib, depicted in Fig. 3(B), in addition to the aforementioned interaction, an extra hydrogen bond was discovered between K1150 and the fluorine atom on the halogenated benzene; π-stacking between F1198’s side chain and the middle pyridine ring was also present. As to the G1202R–crizotinib complex in Fig. 3(C), crizotinib maintains exactly the same binding mode as in the WT and the replaced R1202 uses its long chain to provide extra contact to secure the bound compound. In Fig. 3(D), the L1198F/G1202R–crizotinib complex contains all the interactions that occur in the WT-crizotinib complex, and the π-stacking from F1198’s aromatic ring and the long side chains of R1202 and K1150 add hydrophobic stabilization to the inhibitor.

Listed in Table 1 are the calculated binding free energies determined using the MM/GBSA method for the four studied complexes. Following the structural observation in Fig. 3, the binding affinities of the three mutants are all superior to WT–crizotinib (Δ*G*_bind_ = −16.56 kcal/mol). Notably, the Δ*G*_vdw_ column shows in both L1198F and G1202R mutants the mutated residues provide extra hydrophobic interaction (i.e., ΔΔ*G*_vdw_ = −46.38 − (−41.98) = −4.4 kcal/mol for L1198F relative to WT and ΔΔ*G*_vdw_ = −45.21 − (−41.98) = −3.23 kcal/mol for G1202R relative to WT). Furthermore, these extra hydrophobic interactions are synergized in the L1198F/G1202R–crizotinib complex which has ΔΔ*G*_vdw_ = −48.42 − (−41.98) = −6.44 kcal/mol. Although the rank of the binding free energies agree with the structural characteristics drawn in Fig. 3 where the mutated amino acids F1198 and/or R1202 boost binding affinity, it does not follow the trend of the experimentally measured IC_50_ values in the rightmost column. In the following we adopted kinetic point of view to explain the drug resistance by G1202R.

**Table 1:** Binding free energies calculated using MM/GBSA for crizotinib bound to ALKs. All energies are in kcal mol^−1^.

Model	∆*G*_vdW_	∆*G*_ele_	∆*G*_polar, sol_	∆*G*_nopolar, sol_	∆H_bind_	−T∆S_a_	∆*G*_bind_	IC_50_ (nM)_b_
WT-crizotinib	−41.98	−10.34	20.97	−5.00	−36.35	−19.79	−16.56	20
L1198F-crizotinib	−46.38	−18.91	28.29	−5.31	−42.32	−22.09	−20.23	0.4
G1202R-crizotinib	−45.21	−9.34	21.17	−5.34	−38.71	−17.13	−21.59	382
L1198F/G1202R-crizotinib	−48.42	−19.36	25.72	−5.67	−47.73	−19.07	−28.66	31

^a^The average entropies calculated based on 3 blocks with 10 snapshots/blocks.

^b^Experimentally observed inhibitory activities^22^.

### Structural variance of the binding pocket entrance

**Figure 4:**
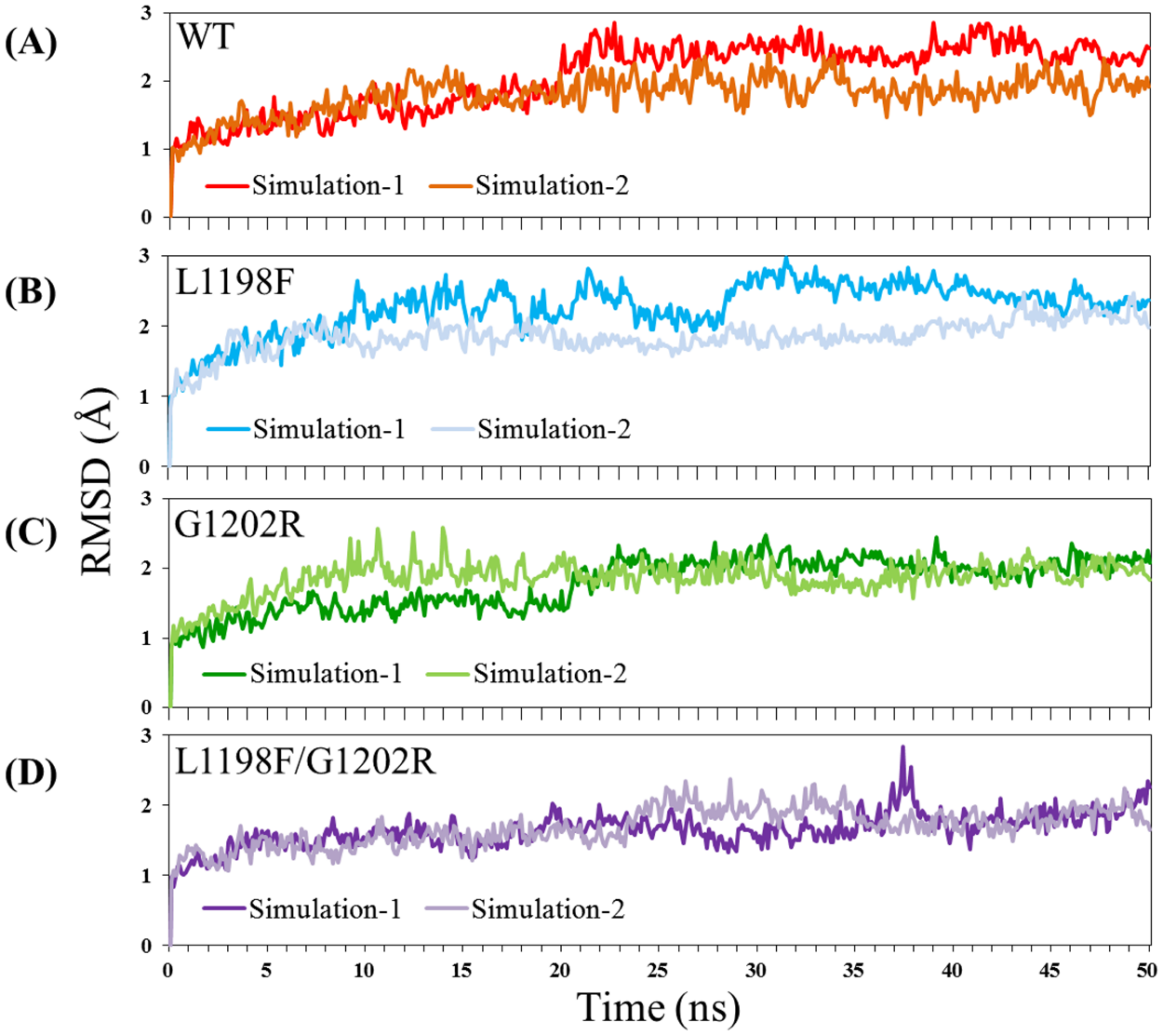
Time evolution of RMSD values of heavy atoms for the four studied apo ALK kinase systems. (**A**) WT, (**B**) L1198F variant, (**C**) G1202R variant, and (**D**) L1198F/G1202R variant.

To monitor the MD trajectory convergence for the four apo ALK systems, the RMSD values of heavy atoms in the production duration were plotted as a function of time in Fig. 4. Each apo ALK underwent two separate MD simulations to increase the abundance of conformation collection. Except for L1198F/G1202R simulation-1 snaps occurring near 37 ns caused by flexible loops in Fig. 4(D), most of the curves fluctuated with variance within 1 Å. Accordingly, for each apo ALK system, we collected 100 snapshots for structural analysis at 10-ns intervals along the 50-ns trajectory of each of the two separate MD simulations.

**Figure 5:**
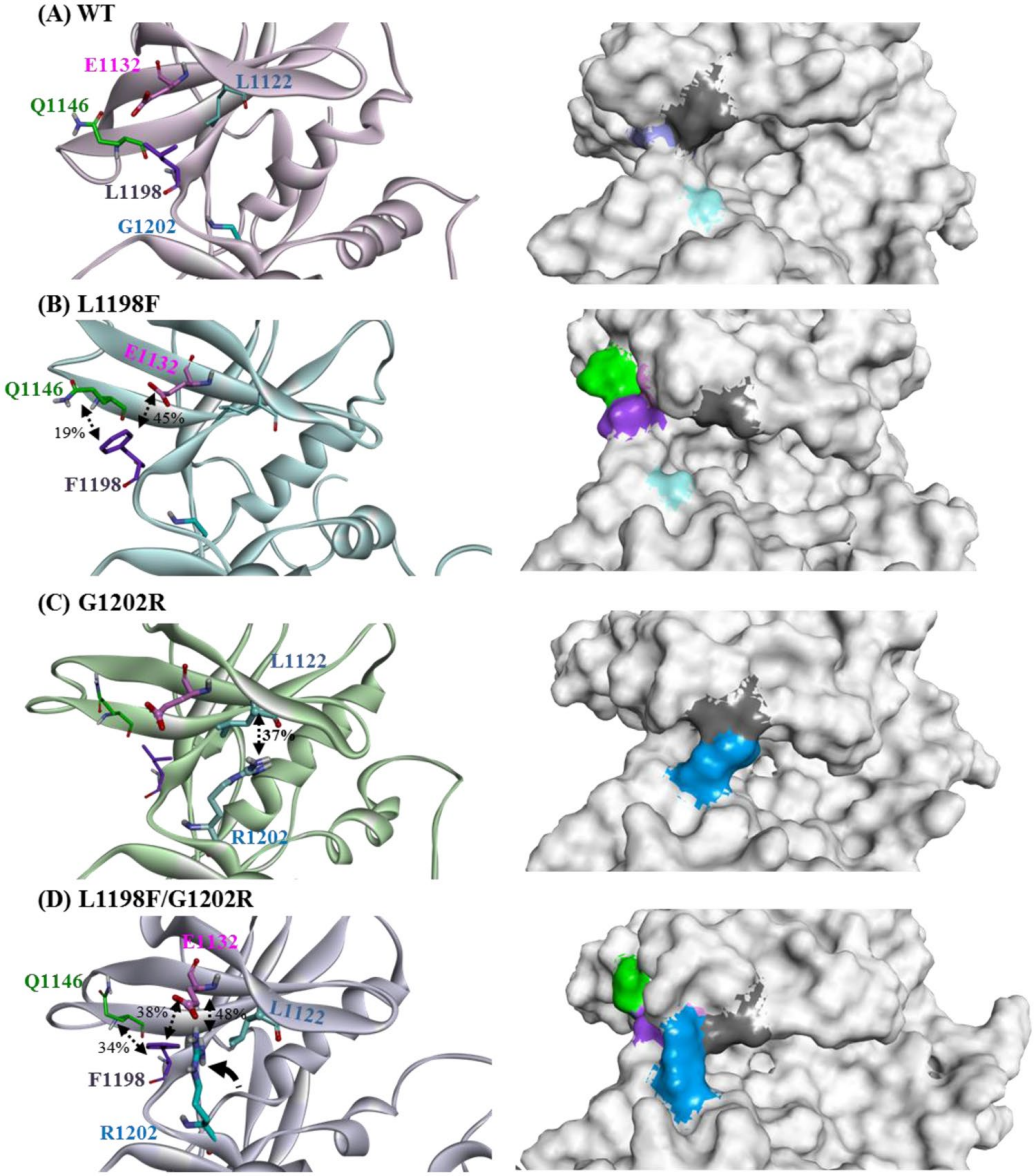
Structural highlights of the crizotinib binding site at the studied apo ALK systems. (**A**) WT, (**B**) L1198F variant, (**C**) G1202R variant, and (**D**) L1198F/G1202R variant. On the surface representations on the right panels, the light-blue/blue surface denotes G1202/R1202, the gray surface denotes L1122, the light-purple/purple surface denotes L1198/F1198, the green surface denotes Q1146, and the pink surface denotes E1132.

Ribbon (in left panel) and surface (in right panel) representations in Fig. 5 provide the structural details centered at the crizotinib binding site on the four ALK systems. The left panel of Fig. 5(A) indicates that in the WT, L1198 is on the hinge and points outward, and the small G1202 sits underneath the crizotinib binding pocket. Thus, the crizotinib binding site is accessible as indicated in the surface representation given in the right panel. As for the L1198F mutant, the left panel of Fig. 5(B) displays that F1198’s bulky hydrophobic side chain still points outward (behaving similarly to L1198 in the WT) and is anchored from the top by Q1146 (on the β3 strand) and E1132 (on the β2 strand) through electrostatic interactions, a weak hydrogen bond with an occupancy of 19% (between the F1198 backbone NH group and the Q1146 backbone O atom), and an electrostatic π-anion interaction with an occupancy of 45% (between the F1198 benzene ring and the E1132 OE1/OE2 atom). Accordingly, the entrance for the crizotinib binding pocket remains clear, and the formation of two hydrogen bonds from hinges E1197 and G1202 toward the pyridine-2-amine ring moiety to secure the inhibitor is still feasible. The right panel of Fig. 5(B) illustrates that the cleft for crizotinib binding is available, and moreover, the cleft is wider than that of the WT, indicating some elevated ease in accommodating crizotinib. As for the G1202R mutant depicted in the left panel of Fig. 5(C), the elongated side chain of R1202 flips upward to interact with L1122 (on the β1 strand) through the hydrophobic interaction with their aliphatic long chains and a weak hydrogen bond (between the NH group of the R1202 side chain and the backbone O atom of L1122) with 37% occupancy. The β1 and β2 strands are elements of the P-loop that shield the ATP–crizotinib binding site. Accordingly, the right panel of Fig. 5(C) depicts that the entrance of the crizotinib pocket is blocked by the L1122–R1202 linkage. This explains how the inhibitory activity of crizotinib in G1202R is greatly reduced — crizotinib has difficulty getting in the binding site. Figure 5(D) presents how F1198 alleviates the drug resistance caused by R1202 in the L1198F/G1202R mutant. As indicated in the left panel, one hydrogen bond is formed between the F1198 backbone NH group and the Q1146 backbone O atom with 34% occupancy, and the π-anion interaction between F1198 and E1132 pulls down the β3 and β2 strands to introduce a linkage between E1132 and R1202 by means of electrostatic interaction. The right panel of Fig. 5(D) illustrates a conformation with the R1202–E1132 salt bridge where R1202’s long side chain tilts to the upper left and leaves the crizotinib binding pocket accessible; although the entrance opening is not as wide as in L1198F as depicted in the right panel of Fig. 5(B), it is similar to that in the WT as displayed in the right panel of Fig. 5(A).

**Figure 6:**
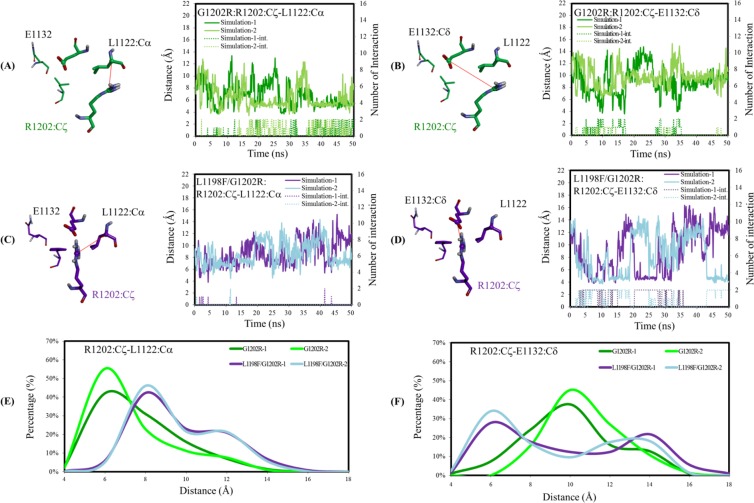
Structural analyses on the R1202 residue in the G1202R and L1198F/G1202R mutants. In (**A**–**D**), separation distance is represented by solid curves with the left-side y-axis reading and interaction count represented by dotted lines with the right-side y-axis reading. (**A**) Distance between R1202 Cζ atom and L1122 Cα atom in the G1202R mutant. The R1202–L1122 interaction is also counted. (**B**) Distance between R1202 Cζ atom and E1132 Cδ atom in the G1202R mutant. The R1202–E1132 interaction is also counted. (**C**) The same monitored items as in (**A**), but for the L1198F/G1202R mutant. (**D**) The same monitored items as in (**B**), but for the L1198F/G1202R mutant. (**E**) Distribution of the separation distance between R1202 Cζ and L1122 Cα atoms. (**F**) Distribution of the separation distance between R1202 Cζ and E1132 Cδ atoms.

To acquire more evidence of how the entrance opening in G1202R and in the L1198F/G1202R mutants are affected by the orientation of the R1202 long side chain, we monitored the R1202–L1122 and R1202–E1132 separation distances. The solid lines in Fig. 6(A) depict the separation distance fluctuation between the R1202 Cζ atom and the L1122 Cα atom in two independent MD trajectories of G1202R. Although the distance could reach a maximal value of 12 Å, it was more likely to remain under 6 Å, which is consistent with the aforementioned hydrogen bond formed between the R1202 side chain NH group and the L1122 backbone O atom. Illustrated in Fig. 6(B) is the separation distance between R1202 Cɛ atom and E1132 Cδ atom of G1202R. Because most of the distance is greater than 8 Å, the electrostatic interaction between the two charged ends rarely occurred. The separation distance between the R1202 Cζ atom and the L1122 Cα atom was also monitored in the L1198F/G1202R mutant, and plotted in Fig. 6(C) showing the R1202 is away from the L1122. Figure 6(D) depicts the separation distance between R1202 Cɛ atom and E1132 Cδ atom, populating between 4 to 15 Å and having a high likelihood of falling under 8 Å, which is consistent with the observation of the aforementioned R1202–E1132 salt bridge. To summarize, when G1202 is replaced by arginine with a long side chain, R1202 points upward and may fluctuate between L1122 (to the upper right and toward the P-loop) and E1132 (to the upper left and toward the hinge). In the G1202R mutant, R1202 more often remains close to L1122, as indicated by the two green curves in Fig. 6(E) that present the high population occurring at a short distance, instead of staying close to E1132, as the two green curves in Fig. 6(F) that present the high population occurring at a long distance illustrate. In the L1198F/G1202R mutant, R1202 movement is rather flexible, but tends to remain close to E1132 instead of L1122. To be more specific, as depicted in Fig. 6(E), the two purple lines for the R1202–L1122 distance in the L1198F/G1202R mutant shift to a longer distance range, compared with the two green curves for G1202R. In Fig. 6(F), the two purple curves peak at 4 Å (higher peak) and 12 Å (lower peak), indicating that R1202 spends some time near and some time away from E1132, and the salt bridge formed between them is likely on and off. Once the salt bridge is formed, R1202 is pulled leftwards to the hinge side leaves the entrance open.

Accordingly, the rankings of the binding pocket accessibility for the four studied ALKs from high to low are the L1198F mutant, the WT, the L1198F/G1202R mutant, and the G1202R mutant, which are consistent with their respective IC_50_ values: 0.4 nM for L1198F, 20 nM for WT, 31 nM for L1198F/G1202R, and 382 nM for G1202R. Moreover, it is likely that the association rate constants (*k*_on_) of these four ALKs follow the same rankings indicating L1198F mutant with an accelerated crizotinib-ALK binding process in comparison with the WT, whereas G1202R mutant with an decelerated binding process.

### Dissociation pathway analysis using PMF

**Figure 7:**
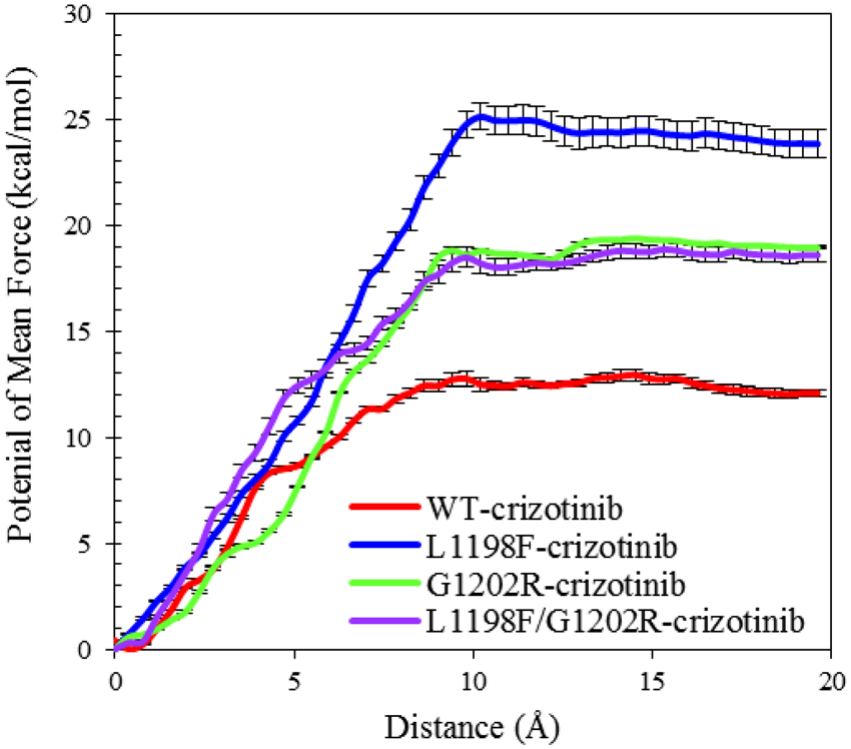
PMFs for crizotinib leaving ALK along the ATP-pocket dissociation pathway for crizotinib-bound ALKs in WT, L1198F mutant, L1202R mutant, and L1198F/G1202R mutant. The PMF curves derived from last 4 ns (7~10 ns) simulation results were convergent and were chosen for comparison. The error bars correspond to the standard error of last 4 ns results for every window.

For many biological events that include protein-ligand association and dissociation and require microseconds or longer to complete, it is difficult to monitor the process with conventional MD simulations. Therefore, enhanced sampling methods such as adaptive biasing force^45^, steered molecular dynamics simulation^46,47^, and umbrella sampling^48^ are ideal tools to overcome the timescale problem. Herein, to investigate the unbinding kinetics, umbrella sampling was used to compute the free energy profile along the dissociation pathway of crizotinib escaping from the ATP pocket of the WT, L1198F, G1202R, and the L1198F/G1202R mutants. As plotted in Fig. 7, the barrier height of L1198F (24.38 kcal/mol at 9.8 Å separation distance) is higher than the other three — 18.98 kcal/mol at 12.9 Å for G1202R, 18.49 kcal/mol at 13.3 Å for L1198F/G1202R, and 12.49 kcal/mol at 9.4 Å for WT. This implies that the L1198F mutant binds crizotinib for the longest residence time among the four. By contrast, the WT has the shortest residence time, and the residence time for G1202R and for L1198F/G1202R are intermediate. The PMF convergence of the four systems is given in Fig. S2 in Supporting Information.

## Conclusion

The ALK G1202R is analogous to the MET G1163R and ROS1 G2302R mutations that are also notorious for crizotinib resistance in NSCLC^49,50^. G1202 is a solvent-front residue and therefore when it is replaced with arginine, the orientation of the R1202 long side chain decisively regulates the accessibility of crizotinib, as determined in our structural analysis of the four apo ALK structures. Figure 5(C) illustrates that in the G1202R mutant R1202 points toward the P-loop and gets fastened by L1122, which seriously narrows the ATP channel opening. However, in Fig. 5(D) for the L1198F/G1202R mutant, R1202 points toward the hinge segment and interacts with E1132, which results in the ATP channel entrance experiencing less effect. It has been reported that the mutation G1202R also confers high-level resistance to alectinib, which was developed to be a more potent ALK inhibitor to combat various crizotinib-resistant ALK mutations such as L1196M and C1156Y^51–53^. Therefore, it is likely that the R1202 residue employs the same mechanism to cause drug resistance on type I kinase inhibitors, associating ALK kinase through the ATP channel.

**Figure 8:**
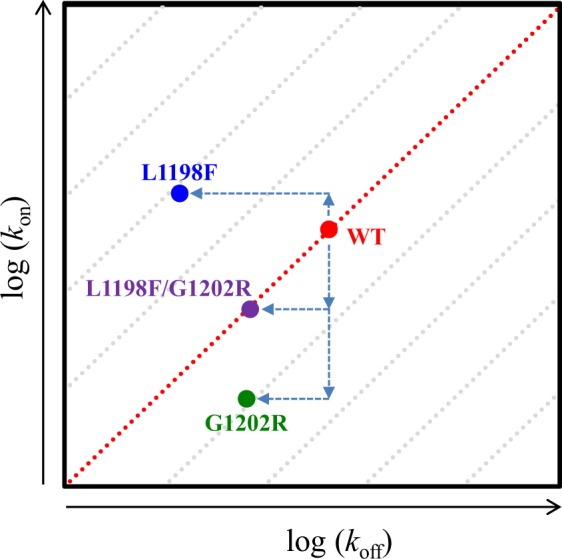
The kinetic profiles of crizotinib bound in the four studied ALKs presented in an on-off-rate map. Each diagonal line is an iso-affinity line where points have an equal *k*_off_/*k*_on_ ratio.

To summarize our findings for the differential crizotinib sensitivity in the ALK mutants carrying G1202R and/or L1198F, instead of using the calculated binding free energies, we focused on the kinetic profiles of crizotinib binding to the four ALKs plotted onto the on-off-rate map in Fig. 8. The map is plotted with an association rate (*k*_on_) versus a dissociation rate (*k*_off_) where the parallel diagonal lines represent iso-affinity lines^54^. Points distributed alone a given iso-affinity line come with the same ratio of *k*_on_ and *k*_off_ values; strong affinity binding events appear in the upper left corner (with high *k*_on_ and low *k*_off_ values) whereas the weak affinity binding events appear in the lower right corner (with low *k*_on_ and high *k*_off_ values). Compared with the WT, the L1198F mutant possesses a larger *k*_on_ value and a smaller *k*_off_ value because the binding channel is more accessible for association (Fig. 5(B)) and the boundary height is higher for dissociation (Fig. 7). Altogether, these effects place the L1198F point to an upper iso-affinity line with an improved drug efficacy relative to the WT’s. As to the G1202R mutant, the shrunken ATP channel opening greatly decreases *k*_on_ (Fig. 5(C)) and the dissociation boundary height slightly higher than that in the WT (Fig. 7) decreases *k*_off_. There two effects move the G1202R point to a lower iso-affinity line with a decreased drug efficacy. In the L1198F/G1202R mutant, *k*_off_ is slightly reduced with a similar decreased magnitude as in G1202R (due to the similar barrier height for both G1202R and L1198F/G1202R mutants in Fig. 7) and *k*_on_ is also decreased, as indicated by the entrance opening width appearing slightly narrower than the WT (Fig. 5(D)). The two effects offset each other and consequently L1198F/G1202R point stays in the same iso-affinity line in which the WT is located.

Lately there have been several theoretical studies centering on the drug resistance mechanisms caused by mutations in ALK^45,55,56^. Sun *et al*. studied three crizotinib-resistant mutants including L1152R, G1202R, and S1206Y by comparing their PMFs with WT’s^45^. They calculated crizotinib binding free energies and concluded structural difference of the four ALK-crizotinib complexes. Entropy influence introduced by P-loop, which is in close contact of the bound crizotinib, was discovered. He *et al*. studied alectinib-bound WT, I1171N, V1180L, and L1198F complexes, where these three mutants are alectinib-resistant^55^. Binding free energies and complex structural stability were evaluated to elucidate the drug resistance mechanisms. Li *et al*. studied different affinities of crizotinib and lorlatinib in WT, and three mutants covering L1198F and/or C1156Y^56^. They simulated crizotinib-, lorlatinib-, and ATP-bound ALKs and discussed how the replaced amino acid residue(s) affect the binding pocket and pinpointed the H1124 plays an essential role. In this presented work, we carried out simulations on WT and three MTs covering L1198F and/or G1202R. Both apo and crizotinib-bound ALKs were simulated. With the apo ALK data, we examined the accessibility of the binding site entrance and further ranked their *k*_*on*_ values; with crizotinib-bound ALKs, we compared their binding free energies and conducted PMF calculations to rate their *k*_*off*_ values. We believe the concluded comparison made for the ratios of *k*_*on*_/*k*_*off*_ highlights the novelty of this study.

We made findings concerning the structural and kinetic interplay of ALK and crizotinib, and hopefully these results can be used to assist the rational design of ALK inhibitors to conquer the problem of mutations.

## Supplementary information

Molecular Modeling of ALK L1198F and/or G1202R Mutations to Determine Differential Crizotinib Sensitivity
