# Supplementary material for: Molecular Modeling of ALK L1198F and/or G1202R Mutations to Determine Differential Crizotinib Sensitivity
Source: Sci Rep. 2019 Aug 6;9:11390. doi: 10.1038/s41598-019-46825-1 (PMC6684801; doi:10.1038/s41598-019-46825-1)
Supplement: Supplementary file 1 — Molecular Modeling of ALK L1198F and/or G1202R Mutations to Determine Differential Crizotinib Sensitivity [file 41598_2019_46825_MOESM1_ESM.docx]

**Molecular Modeling of ALK L1198F and/or G1202R Mutations to Determine Differential Crizotinib Sensitivity**

Yu-Chung Chuang^1^, Bo-Yen Huang^1^, Hsin-Wen Chang^1^, Chia-Ning Yang*^1,2^

^1^ Department of Life Sciences, National University of Kaohsiung, Kaohsiung, Taiwan

^2^ Scientific Multi-Simulation Center, National University of Kaohsiung, Kaohsiung, Taiwan

**Supplementary Information**


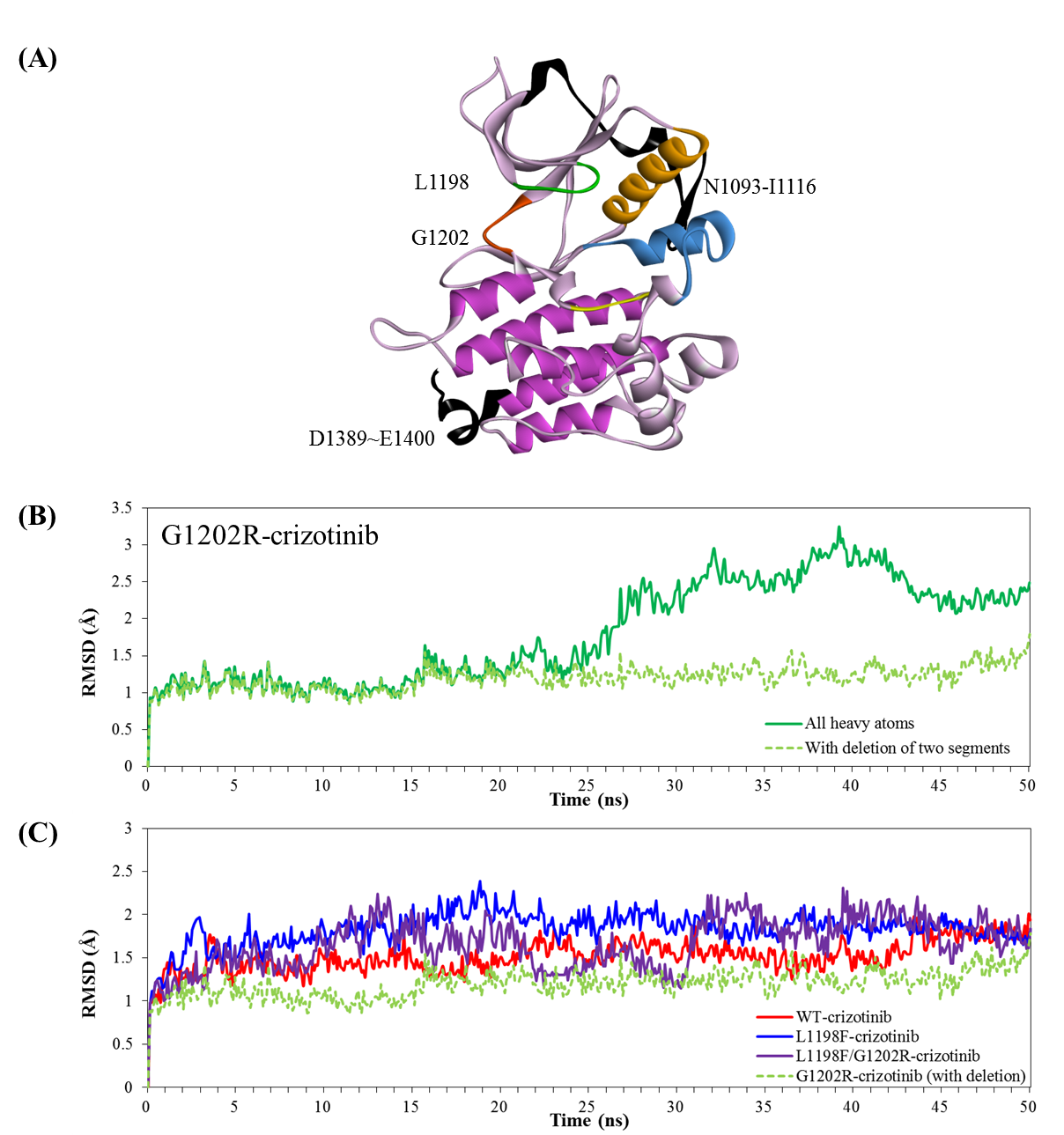


**Figure S1** Insights into the highly flexible RMSD curve of G1202R in Figure 2(A). (A) An ALK structure showing the two mobile segments, colored in black, in the N- and C-terminus responsible for enlarging the RMSD values in G1202R-crizotinib complex. (B) A comparison made for the G1202R-crizotinib complex where the two mobile segments are included (in solid line) or deleted (in dashed line). (C) RMSD curves plotted for the four ALK-crizotinib complexes where G1202R-crizotinib curve uses the deleted structure and agrees with the other three curves.


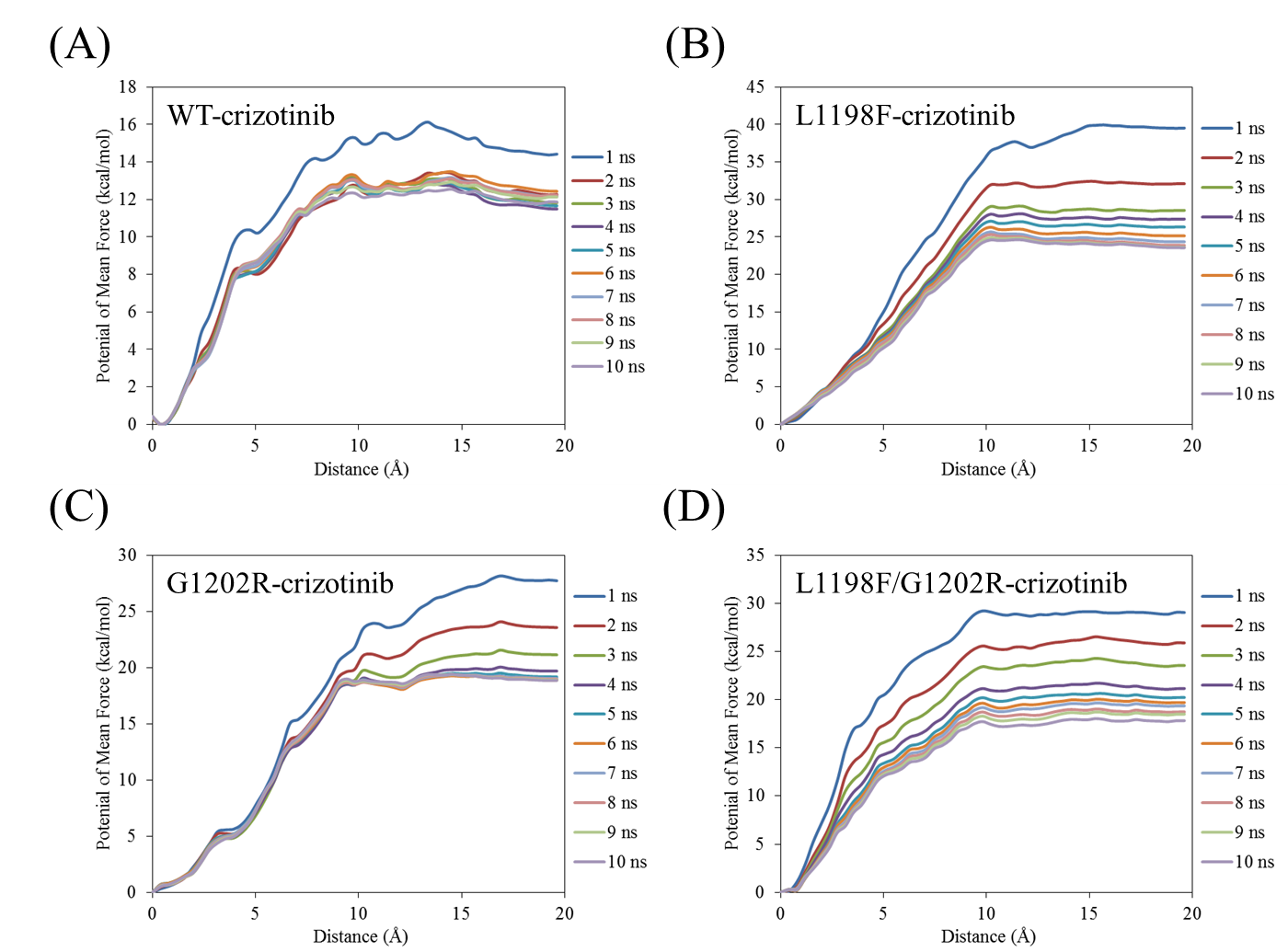


**Figure S2** Convergence of the PMFs calculated by 17-ns umbrella sampling for (A) WT-crizotinib, (B) L1198F-crizotinib, (C) G1202R-crizotinib, and (D) L1198F/G1202R-crizotinib.
